# Cross-sector, sessional employment of pharmacists in rural hospitals in Australia and New Zealand: a qualitative study exploring pharmacists’ perceptions and experiences

**DOI:** 10.1186/s12913-014-0567-4

**Published:** 2014-11-13

**Authors:** Amy CW Tan, Lynne M Emmerton, H Laetitia Hattingh, Adam La Caze

**Affiliations:** Pharmacy Australia Centre of Excellence (PACE), School of Pharmacy, The University of Queensland, Level 4, 20 Cornwall St, Woolloongabba, Queensland 4102 Australia; School of Pharmacy, Curtin University, Building 306, Kent Street, Bentley, Western Australia 6102 Australia

**Keywords:** Clinical pharmacy, Hospital, Medication management, Pharmacist, Rural, Sessional, Service models, Qualitative

## Abstract

**Background:**

Many rural hospitals in Australia and New Zealand do not have an on-site pharmacist. Sessional employment of a local pharmacist offers a potential solution to address the clinical service needs of non-pharmacist rural hospitals. This study explored sessional service models involving pharmacists and factors (enablers and challenges) impacting on these models, with a view to informing future sessional employment.

**Methods:**

A series of semi-structured one-on-one interviews was conducted with rural pharmacists with experience, or intention to practise, in a sessional employment role in Australia and New Zealand. Participants were identified via relevant newsletters, discussion forums and referrals from contacts. Interviews were conducted during August 2012-January 2013 via telephone or Skype™, for approximately 40–55 minutes each, and recorded.

**Results:**

Seventeen pharmacists were interviewed: eight with ongoing sessional roles, five with sessional experience, and four working towards sessional employment. Most participants provided sessional hospital services on a weekly basis, mainly focusing on inpatient medication review and consultation. Recognition of the value of pharmacists’ involvement and engagement with other healthcare providers facilitated establishment and continuity of sessional services. Funds pooled from various sources supplemented some pharmacists’ remuneration in the absence of designated government funding. Enhanced employment opportunities, district support and flexibility in services facilitated the continuous operation of the sessional service.

**Conclusions:**

There is potential to address clinical pharmacy service needs in rural hospitals by cross-sector employment of pharmacists. The reported sessional model arrangements, factors impacting on sessional employment of pharmacists and learnings shared by the participants should assist development of similar models in other rural communities.

## Background

Pharmacists play a major role in promoting optimal medication management. In a hospital setting, pharmacists are involved in the review of patients’ medications, medication reconciliation, medication dispensing, provision of medication information, staff education and quality management relevant to medications [1]. Pharmacists’ clinical services, involving prescription review and therapy recommendations (initiation, dosage changes, cessation or substitution of treatments), have been documented to improve symptom control, increase treatment efficacy and reduce adverse events [2,3]. Studies have demonstrated that a pharmacist’s provision of medication information and consultation can have positive impacts on patients’ self-management of their medications [4,5]. In addition, the value of pharmacists’ discharge liaison and specialist medication review services, aiming to reduce medication misadventures and related hospitalisations, has been recognised [3,4].

Despite this evidence, there is sub-optimal provision of medication management activities, such as inpatient medication reviews, medication reconciliation and medication counselling, in rural Australian hospitals [6-9]. This is mainly a result of rural pharmacist shortages, as rural areas pose a challenge to recruit and retain health practitioners, and to sustain adequate health services locally [10]. Many rural hospitals do not have a pharmacist employed on-site [11,12], referred to as ‘non-pharmacist hospitals’ in this paper. Although the roles of registered nurses in these rural hospitals have been extended to provide medication supply and basic medication information services, there have been reports of clinical and logistical challenges faced by the nurses, and the need for additional medication management support has been identified [6,7,9,11,13].

A strategy adopted by some rural hospitals is the contracting of existing private healthcare providers to deliver services that the hospital is unable to provide internally [10,14,15]. Such contracting of non-permanent or continuing staff, referred to as ‘sessional’ [7,14] services in this paper, enables better utilisation of rurally available skills and workforce [10,14]. The term ‘cross-sector’ in this paper refers to a contractual agreement between sectors, e.g. between public and private. A common example is a rural general practitioner providing primary care services in private practice and being employed as a visiting medical officer to provide services at the local hospital [10,14]. Similar sessional employment has been reported for certain allied health professionals, such as optometrists, dietitians, podiatrists, occupational therapists and physiotherapists [7,14,15].

Despite existing sessional models, there is a paucity of research on similar models involving pharmacists. A scoping study on medication management issues in a rural community in Australia identified opportunities for sessional services involving pharmacists [8,16]. However, there are no specific studies exploring the extent of sessional pharmacist services, employment arrangements, or challenges and enablers, which prompted the current research. Another driver for this study was the emerging trend for, but limited studies on, cross-sector, public-private partnerships to optimise resources in provision of health services [17,18].

The aim of this study was to explore sessional employment models involving pharmacists providing services to rural hospitals. The objectives were to identify:

- Enablers to the establishment, implementation and operation of the sessional models, and
- Challenges and coping mechanisms adopted or suggested by pharmacists involved.

Pharmacists’ insights into the experience of delivering (or planning to deliver) these services may assist development of sessional pharmacist models in other rural communities in Australia, and potentially overseas.

## Methods

Ethical approval for data collection was obtained from The University of Queensland’s School of Pharmacy Ethics Committee (2012/9). Qualitative methods were deemed most appropriate to explore and contextualise the sessional models [17,19]. Purposive sampling [20] targeted pharmacists with experience in providing pharmacy services on a sessional basis to non-pharmacist hospitals, although pharmacists working towards sessional pharmacy services were also recruited to gain insight into how they were planning to develop and implement this model of employment.

As there was no database listing Australian pharmacists employed in a sessional capacity, the demographics and statistics around sessional pharmacists were not known. Hence, a *de novo* approach to sampling was undertaken using a range of strategies. This included advertising the study in Australian professional newsletters and forums (Pharmaceutical Society of Australia electronic newsletters and rural forum, *Guild Forefront*, The Society of Hospital Pharmacist of Australia electronic newsletters, *Pharmacy Daily*, *Pharmacy News*, Information to Pharmacists ‘*i2P*’, National Rural Health Alliance electronic newsletters, *Australian Journal of Pharmacy* and *Auspharm* forum), and forwarding an ‘Invitation to Participate’ to pharmacy contacts. Recruitment was conducted by AT and continued until all identifiable potential participants had been approached and the sampling pool had been exhausted.

Consultation with New Zealand academic researchers indicated that this model of employment had been implemented in New Zealand. The Australian sample was supplemented with New Zealand pharmacists identified as having experience with sessional pharmacy employment, with a view to gain further insights into the implementation of their sessional services and to allow some comparison of factors facilitating this type of employment. This was considered to add richness to the data and further inform future sessional initiatives. Recruitment in New Zealand was facilitated by two academic contacts at the School of Pharmacy, University of Otago, using hospital registers. Identified New Zealand sessional pharmacists were then contacted by a colleague in New Zealand to confirm the nature of their service provision, before being invited to participate in the study. Pharmacists with verified sessional roles were subsequently contacted by AT to participate in the study.

All pharmacists contacted were informed about the study (with the provision of verbal and a written information sheet), had a suitable interview time arranged and were asked to provide verbal consent at the start of the interview. All interviews were conducted by author AT via either telephone or Skype™, between August 2012 and January 2013. Interviews ranged from 40 to 55 minutes, and each participant was sent a AUD$50 or NZD$50 gift card as a token of appreciation.

Interview topics aimed to elicit in-depth insight into scopes of service, contractual arrangements, and factors impacting on the sessional employment models, such as workforce, funding, inter-professional relationships and clinical skills. Interviews were based on a semi-structured guide informed by literature on government partnerships and integrated healthcare services [15,21,22], research describing rural health services [7,10,23-25], and communication with informants involved in health service administration and rural pharmacy services. The following questions are extracted from the interview guide:

1. What do you believe was the underlying reason for setting up this sessional service?
  - What are/were the presenting problem(s)/issue(s) that might have prompted the introduction of sessional employment for the pharmacist?
  - Which health professionals are/were involved? How?
  - What are/were the expected outcomes of this arrangement?
  - What do you think you’re adding (or you’ve added) to the community in this role?
2. Let’s discuss how your model operates. What are/were the employment arrangements like?
  - What are/were your roles/responsibilities as the sessional pharmacist?
  - Who pays (paid) which parts of your position? How did this pay reach you (e.g. single pay packet, periodic invoice)?
  - What are/were your working hours/FTE like?
  - For how long are/were you contracted?
3. What do you think are/were the enablers or challenges for this model?
  - How do you feel about your workload?
    • Is/was there support from other pharmacy staff?
    • Is/was there assistance from other healthcare providers?
  - Are/were there any training, mentoring or clinical support for you to undertake this role?
  - What about funding or financial support for the model?
  - How would you describe the relationships between you as the practising pharmacist and other healthcare providers?
  - How would you describe the reception for your service?
4. How do you cope with (the challenges described)? What support did you receive/do you need?
5. How much satisfaction are you getting out of this role?
  - If you’ve moved on to something else, do you have any plans for other pharmacists to take over?
  - Are there any other changes/enhancements you would like to suggest?

Relevant parts of the recorded interviews were transcribed verbatim, excluding identifiable details. Thematic analysis [26] was concurrent with data collection. This enabled testing of emerging concepts, themes and categories against subsequently-collected data, and allowed for adjustments to be made to interview topics in accordance with qualitative methodology [27]. Author AT performed the initial qualitative data analysis by identifying key themes and divergence within each interview topic, and extracted data from transcripts to support the themes. These themes and relevant data were discussed with the other authors, who clarified, verified and refined the findings. Final confirmation of themes was achieved through consensus of all authors. These themes are presented in the following sections, supported by quotations.

## Results

**Figure 1:**
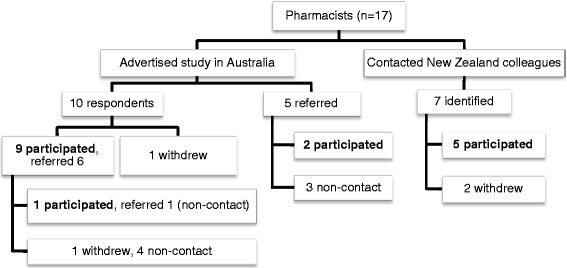
**Recruitment of participants.** Those who did not participate either did not respond to repeated Invitations to Participate (non-contact) or initially responded but failed to follow up (withdrew).

**Figure 2:**
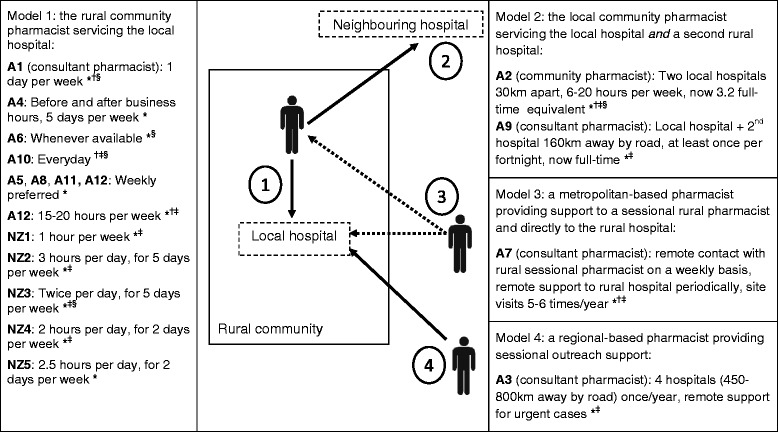
**Overview of sessional models based on participants’ descriptions (models are divided into four general types).** Most models were ongoing services at the time of study. Participants A4, A6 and A10’s model was discontinued. Participants A5, A8, A11 and A12 were working towards sessional pharmacist support in their community. Some pharmacists practising in Australia have attained additional accreditation and were employed as a private practitioner/consultant providing specialist medication review services to patients in the home and aged care facilities. Sessional roles/services provided include: *inpatient medication review, discharge liaison, general medication information support; ^†^in-service education and medication advisory; ^‡^administrative support (accreditation, therapeutic drug review, clinical governance); ^§^pharmaceuticals distribution or dispensing.

Figure 1 provides an overview of recruitment for this study. Seventeen pharmacists participated in the interviews, 12 practising in Australia (designated as A1-A12) and five practising in New Zealand (designated as NZ1-NZ5). Of the 17 pharmacists (six males, 11 females), five had sessional experience from previous employment: two models (A2, A9) had expanded to full-time equivalent appointments, and three models (A4, A6, A10) were discontinued. Eight participants (A1, A3, A7, NZ1-NZ5) were providing sessional services at the time of study. The four remaining participants (A5, A8, A11, A12) were seeking support for rural non-pharmacist hospital(s) in their respective community, and discussed their proposed sessional services. Figure 2 illustrates sessional models described by all 17 participants.

To maintain confidentiality, participants are not associated with their age, location of practice or employers. Apart from three participants who had at least five years of practice experience, the majority of the participants had been practising pharmacists for longer than 10 years, up to approximately 30 years. The majority of participants were local members of their communities, whereas participants A3-A8 had work experience in rural communities. Participants were recruited from a number of jurisdictions in Australia (South Australia, Western Australia, New South Wales and Queensland) and New Zealand (North Island and South Island).

Twelve participants were practising in towns with fewer than 5,000 residents, three participants in towns with approximately 5,000-10,000 residents, and two participants in regional centres comprising 13,000 and 75,000 residents, respectively. All identified hospitals were the only hospitals in these towns, with the exception of participant A10, who serviced a private hospital in a two-hospital town. Most of the hospitals were publicly funded. Hospitals serviced by participants NZ1 and NZ2 were partially privately owned. As anticipated in rural areas, almost all hospitals had an inpatient capacity of fewer than 50 beds, and the majority relied on visiting medical practitioners to service the hospitals. The majority of participants indicated that their services aimed to address medication management needs at the hospitals, and the study did not identify a minimum population or hospital size warranting sessional employment. Ideal conditions for effective sessional models were not identified, although commonalities were evident in the data.

### Perceived value of sessional pharmacists’ services

Sessional employment enabled pharmacists to provide inpatient services, in-service education, medication advisory support, pharmaceutical distribution support, and dispensing services. All participants perceived their sessional roles to be of clinical value through direct patient care and indirect input into medication management processes at the hospitals. In addition, they asserted that the sessional roles provided additional support to hospital nurses who were perceived to be challenged by undertaking extended medication roles beyond their traditional scope of practice. The sessional models were viewed by all participants as a practical and sustainable initiative to increase medication management support in rural hospitals, by involving the rural pharmacist, without relying on outreach services.

Key enablers for establishing the service included the hospital’s recognition of its value and acceptance among staff. In the absence of outcomes data, these facilitated justification for funding allocation for the sessional service:

“*It was really quite far-sighted of the regional director to approach me… [the director] knew me in my role with doing [medication management reviews], and they knew that they needed a pharmacist… without [the director] recognising that, there wouldn’t have been any access to that sessional work.*” (A9)

### Collaboration and communication

The various hospitals’ management structures provided different levels of support and recognition for a pharmacist’s involvement at the hospital, which variably challenged the establishment of a sessional service model. Participants commented that recognition of a pharmacist’s role might have been limited by the lack of experience working with a pharmacy-trained colleague. This was perceived as a challenge to the integration of a pharmacist and the establishment of the sessional service at the rural hospital.

Many participants suggested that the level of recognition could be increased through ongoing collaboration, communication and rapport building. Several participants were able to further demonstrate the value of their involvement through established collaborations within their community. This resulted in increased recognition for a pharmacist’s role, and subsequently, expansion of service scope:

“*I was able to demonstrate that if you had good pharmacy service, you actually save nursing time… That results in better patient care. Initially, [some] doctors were not that accepting… but then the doctors realised it as well.*” (A2)

Some participants added that, based on their experience, it took many years to build rapport and to demonstrate the value of their services:

“*This is something that I’ve worked on for over 10 years now… gaining trust… It’s very much having worked with other people and making them aware that your skills are there.”* (A1)

The importance of continuous engagement was also highlighted:

“*Probably to start with, they wondered whether I’d hang about, because they’d probably seen a lot of people come and go over the time, saying ‘yes, we’ll do these great things for you’, and then because it gets too hard or too expensive, they leave.*” (A3)

### Autonomy at the local level

A common theme was the considerable autonomy and independence in service planning *and* funding allocation within the local district or rural community. This enabled community participation (i.e. hospital management, nurses, doctors and the pharmacist) to formulate and implement the sessional services to address local medication management needs. This was a prominent feature in all New Zealand and several Australian models (A1, A2, A9).

In the cases of participants NZ1 and NZ2, the hospitals were partially funded by a community-based private trust fund, enabling the community to drive local initiatives including their sessional services. Participant NZ3’s hospital was bulk-funded, which enabled flexibility in expenditure, and this allowed the participant to negotiate service specifications and costs directly with the hospital’s management. The services provided by participants NZ4 and NZ5 were supported by the local district health board, which managed public hospitals within the district.

Successful models in Australia (A1, A2, A9) were supported and funded by their local area health service, which also managed public hospitals within the district. A distinct difference between the Australian and New Zealand systems was that the area health service was managed by the broader state/territory health government. Autonomy in service planning and funding allocation thus varied depending on the governance structure in each state/territory. In this study, funding allocation at a local level in participants A1, A2 and A9’s communities appeared to be more flexible and autonomous, as compared to other communities serviced by participants A3, A5, A8, A11 and A12.

The latter participants commented on the financial challenges when funding control was centralised at the state level as opposed to decentralised at the local state health district or rural community level. In these models, participants commented on the difficulty in proposing a business case and in securing funding for pharmacist services. The centralised administration body was considered insensitive to, or unaware of, local issues and service gaps, and therefore, failed to recognise the value of pharmacist-mediated sessional services to address medication management needs:

“*All this funding allocation is controlled by the financial people [i.e. corporate services department within the centralised administration body], and they don’t really necessarily understand [the value of a pharmacist]… They would say, ‘hey, if I’m only able to claim $100 worth of [medications subsidised under the Australian Pharmaceutical Benefits Scheme] in a day and it costs $400 to employ a pharmacist a day, I’m not going to do it.’*” (A5)

Participant A12 added that the lack of recognition of needs and gaps at the local level caused central administrators who review hospital funding budgets and allocation to rely on cost justification based on quantifiable outcomes. Participant A12 highlighted the lack of economic data, and indicated that funds would not be provided if outcomes were not generated, and this could not occur without funding.

### Funds pooling

The availability of funding was crucial to ensure sustainable implementation, despite some pharmacists providing sessional services for little financial reward. Many Australian participants commented on the challenge when there is a lack of funding allocation from state government departments designated to administer and manage the hospital services. This resulted in several participants relying on funds pooled from other sources to supplement the remuneration of the sessional pharmacist position. Two main funds-pooling arrangements were identified.

Participants A7 and A10’s service was supported by a business model associated with pharmaceutical supply and distribution services between the local community pharmacy and the hospital. This provided the pharmacy with sufficient profit margins to cross-subsidise the participants’ sessional services, such as inpatient medication review and medication management advisory activities. Similar arrangements were also identified for participants NZ2 and NZ3, albeit aiming for convenience and holistic service provision rather than an additional financial buffer.

Several Australian models (A1, A2, A3, A7) involved provision of medication management review services to eligible patients in their homes (known as Home Medicines Review) [28] and in government-funded residential aged care facilities (known as Residential Medication Management Review) [29]. These were remunerated by the Australian Federal Government [28,29]. Participants A1, A3 and A7 provided these services at the hospital as part of their sessional services, whereas participant A2 provided them externally to the hospital. Payment for these services supplemented the participants’ sessional employment, reducing reliance on state government funding.

While pooling of funds provided additional means for sessional services, some Australian participants acknowledged limitations relating to eligibility criteria for funding schemes such as the federally-funded medication review services, inconveniences from claiming payments from multiple funding streams, and difficulty balancing between providing additional support and being fully remunerated. Detailed findings and discussion on funding options for pharmacist-mediated sessional services in Australia are reported elsewhere.

### Enhanced recruitment and retention

Participants identified that a successful feature of the model involved having more than one pharmacist within the community for workload management (A1, A2, A7, A10, NZ1, NZ2, NZ3, NZ4, NZ5). A model involving a single pharmacist (A4, A6) servicing both community pharmacy and hospital settings is possible, although not desirable and potentially unsustainable. Participants also commented that sustainability for the model is increased with the employment of a pharmacist that has settled and integrated within the rural community.

Interestingly, some participants discussed opportunities to address the rural pharmacist workforce shortage through the sessional models by increasing workload, and hence, increasing employment opportunities in rural communities to dispense the workload. Some Australian participants, who were independently employed as accredited pharmacists to provide medication management review services to community and aged care patients, commented on the availability of sessional hospital work to complement their consulting services and facilitated their retention in the rural communities:

“*If you can set up a position with income providing hospital services, residential services to nursing homes, and [Home Medicines Reviews] to a certain geographical area, that may help keep one clinical pharmacist in the area with some appropriate employment opportunities.*” (A1)

Recruitment opportunities for non-accredited pharmacists were also identified with increased workload through expansion of community pharmacy services to the hospital. At the time of study, all participants with ongoing sessional roles indicated that they intended to retain their employment and continue with their respective service, as they found it professionally satisfying and career enhancing:

*“There was no financial advantage… I got paid a little bit less by the hospital per hour than I was by the retail pharmacy… [but] I didn’t really want to give up the hospital… you do it for the love as well as the money.” (NZ2)*

*“I feel that’s part of my contribution to helping them provide a topflight service to the people… this allows me to see patients and to practise clinical pharmacy. For me that’s a reward in itself.” (A7)*

### District pharmacy support

Varying opinions were noted around hospital pharmacy skills and experience required to provide sessional services. Some participants commented that experience in hospital pharmacy facilitated establishment and provision of sessional services. Other participants found that general pharmacy practice knowledge and experience were sufficient to provide the needed support at the hospital, and that it was possible to up-skill through professional education and peer learning.

However, one prominent theme was the need for induction and continuing support within the rural district, preferably provided by a regionally or metropolitan-based hospital pharmacist. This was considered valuable particularly for participants in towns lacking pharmacist workforce and relevant peer support. Some participants who received district support were appreciative of the clinical and information support provided by a designated district pharmacist or district pharmacy located at a base hospital. Examples included distribution of hospital policies, updates on medication therapies and related protocols, information on stock expiry or product recalls, and occasional site visits to provide the required technical or clinical support and to assist with implementation of new systems at the hospitals. This support eased adaptation to the hospital setting, as reflected by some participants:

“*From my perspective, you do need a lot of support to start off with… Someone who’s in hospital pharmacy, who’s just checking up, feeding you information – it doesn’t have to be much, it just has to be what you’ve got to deal with.*” (NZ4)

“*There’s different focus involved and different regulations and policies and stuff that you’ve got to remember and think about… You just need some clinical governance and professional debriefing. You need someone you can say to, ‘This is what I did, do you think I did the right thing?’*” (A2)

Other district support received included pharmaceutical distribution and assistance with accreditation and administrative activities, which enabled the sessional pharmacists to focus on provision of inpatient clinical services within the limited contracted time. Some participants also suggested the potential for district support in providing or obtaining locum cover.

It should be noted that the level of district support varied greatly between models; descriptions from New Zealand participants illustrated a larger extent of peer support mechanism. Some Australian participants highlighted the lack of district support systems. These participants resorted to workplace learning and professional education, which was challenging initially and not as ideal as having a district mentor.

Insights from participant A7 suggested that the majority of district pharmacy support could be provided electronically via teleconferencing or videoconferencing, alleviating the need for frequent long distance travel. Participant A7 provided weekly support and feedback via email or telephone to a graduate pharmacist in a rural community, who serviced the local hospital on a sessional basis.

### Service tailoring and flexibility

The variability of the models suggests the need for the arrangements to be flexible. Sessional roles and/or work hours in most working models were tailored based on services required at the hospital, funding availability, local workforce capacity, and skills of the sessional pharmacist. Thus, it was considered impractical for a sessional pharmacist to provide hospital pharmacist services to the level expected in a multi-pharmacist metropolitan hospital, such as those listed in the Society of Hospital Pharmacists of Australia’s *Standards of Practice for Clinical Pharmacy Services* [1].

Some participants commented on the importance of defining the role during early stages of the service to enable prioritisation and tailoring of services:

“*Being clear about the role to start off with is very important, clarifying what’s actually expected of the role – then it’s about building rapport with the staff… and developing that role… [otherwise] you could go crazy and do a whole lot of things and constantly burn yourself out*.” (NZ4)

“*Initially, what I was looking at was a lot of access issues, access to medications… Then I got a bit more time, then got more systems going, then I started providing clinical services, reviewing patients’ notes, and doing patient counselling.*” (A2)

On clarifying the sessional role, most participants then highlighted the importance of flexibility in the role in accordance to service needs and workload:

“Y*ou’ve only got two hours to be there, and you might come across things that you really need to spend more than two hours to deal with. So that is challenging… maybe there’s a means to do more hours when needed, but take time off [on quieter days], and balance that somehow.*” (A9)

This flexibility in service provision was perceived to be appreciated by hospital staff. It also assisted the participants to cope with the limitations of their respective service, which facilitated its longevity.

## Discussion

This is the first known published study exploring sessional employment models involving pharmacists across Australia and New Zealand. Findings from this study add to the limited literature on cross-sector, sessional employment in the health industry, with a rural focus [10,14-16]. While data were limited to 17 pharmacist-participants, the strength of this study lies in the in-depth perspectives obtained through qualitative techniques. Although the models varied, there is evidence from these interviews that sessional employment can be successfully implemented to improve provision of medication management and pharmacy services in non-pharmacist hospitals.

Recognising the value of pharmacists’ involvement was perceived as crucial in establishment of sessional models. This finding is similar to other health services studies highlighting that stakeholder support and partner acceptance were key to success in cross-sector partnerships [17,18,21,22,30], and hence should be addressed early on in model development and implementation. However, the lack of outcomes data and awareness of the value of pharmacists’ involvement presented a challenge for some participants in initial introduction of services. Continuous engagement, collaboration and communication within the local community were presented as some initiatives to enhance service recognition. While joint planning and collaboration have been identified by some health services studies on public-private partnerships [17,18], ongoing and long-term engagement was emphasised in this study. This was considered a crucial factor in a rural setting to promote the community’s readiness for the service [22,24], which was not identified in the previous studies [17,18] potentially attributed to their lack of rural focus.

Funding is a crucial factor in facilitating development and implementation of the service. The discussion on funding of the sessional service raised two key issues. Firstly, the findings showed the importance of service planning and funding autonomy at the local level, provided that the significance of the service is appreciated. This was aligned with published studies supporting decentralisation of governance in enabling the rural community to tailor initiatives based on local needs [21,23,31,32]. The cost-benefit value was not recognised in hospitals with centralised administrative management, which was considered insensitive to or unaware of local issues and service gaps, in line with some published reports [18,22,23,31]. This resulted in difficulty to drive funding allocation for local initiatives such as the sessional pharmacist employment model. Secondly, while localised funding decisions should facilitate the implementation of a sessional service model, the lack of sufficient funding from the designated government department to financially sustain the service was identified as a barrier. However, this study demonstrated the benefits of cross-sector partnerships, enabling pooling of funds to provide financial relief and sustain the service. While similar financial models have been reported in other rural Australian studies [15,22,23,30], this study provides additional insights into funds pooling options for sessional services specifically involving pharmacists, particularly in an Australian practice setting.

An adequate pharmacist workforce was considered important to operate the sessional service. Rural workforce recruitment and retention issues were a recurrent theme in the literature review [7,10,33]. This study demonstrated employment opportunities that may, in part, address rural pharmacist workforce shortages through increased workload and opportunities for career satisfaction, the lack of which have been reported to discourage skilled workforce in rural areas [10,33]. In addition, sessional models may provide opportunities to address oversupply of pharmacists in non-rural areas, currently the case in Australia [33,34]. This employment model creates job opportunities for pharmacists with the potential of facilitating expansion of workforce into rural areas and address workforce maldistribution.

The design of the sessional pharmacist service can impact on the continuity of the service provision. This study highlights two key aspects for consideration when developing a sessional pharmacist service, namely support mechanisms and flexibility, both of which have been reported as factors impacting on rural services and workforce retention [10,23,24,33]. The significance of district support systems and how these were implemented was emphasised in this study; such mechanisms have not been reported in other cross-sector partnership studies [15,17,18,30]. On the other hand, flexibility in the arrangements ensured that the sessional service was tailored around existing resources in the community, a feature in designing integrated models identified in other studies [15,18,21,23,24]. This study provides additional insight into how increased flexibility can help the sessional pharmacist to cope with service provision.

### Limitations

The small sample size limits the generalisation of the findings. However, the similarities shared among participant views indicate that new information would not have been identified with a larger sample. Findings were thus considered sufficiently meaningful and transferrable to other rural communities.

Notwithstanding differences in governance structures and legislation between Australia and New Zealand, practices appeared similar. Hence, this study did not involve a discrete international comparison of service models as initially intended. However, the extension of sampling to New Zealand to include five additional sessional pharmacists added breadth and richness to the data relating to service planning and implementation, as there were only three Australian participants with ongoing sessional roles at the time of the study.

The insights were limited to pharmacists’ perspectives. Future studies could involve insights from other healthcare providers into the feasibility and utilisation of sessional pharmacists to provide medication management support in rural Australian and New Zealand hospitals.

## Conclusions

The reported sessional model arrangements, factors impacting on sessional employment of pharmacists and learnings shared by the participants should assist development of similar models in other rural communities. Given the novelty of cross-sector partnership and employment arrangements in the literature, findings from this research provide a fundamental framework on sessional model arrangements for health service researchers and policy makers to further evaluate the potential of sessional models involving pharmacists. Ideas from this study may also be applicable to other health professionals and health settings, nationally and internationally.
